# Women, Gender, and Mental Health: Analysis of a Case of Multiple Psychiatric Diagnoses and Gender Conditioning

**DOI:** 10.1192/j.eurpsy.2025.2429

**Published:** 2025-08-26

**Authors:** A. Romero Teruel, M. L. del Pozo, C. P. Usaola

**Affiliations:** 1 Psiquiatría, Hospital Dr. Rodríguez Lafora; 2 Psiquiatría, Centro de Salud Mental de Hortaleza (HRYC), Madrid, Spain

## Abstract

**Introduction:**

Gender significantly impacts the diagnosis and treatment of mental disorders. This case shows how being a woman, alongside trauma and violence exposure, shaped a patient’s diagnoses over time, affecting recovery, and culminating in a late-stage lung cancer diagnosis.

**Objectives:**

Analyze how experiences of violence, trauma, and social stigmatization shaped the diagnostic path of a female patient with schizophrenia, multiple hospitalizations, and a fatal outcome, with delayed lung cancer diagnosis despite recurrent emergency visits for respiratory issues.

**Methods:**

We reviewed the clinical course of a 55-year-old woman with schizophrenia. She was admitted to a psychiatric care unit from November 2000 until 2017. In her final years, she was treated for respiratory issues that eventually led to a diagnosis of lung cancer with vertebral metastasis. The late diagnosis underscores the impact of her psychiatric history on care quality.

**Results:**

**Violence and Trauma:**

- The patient experienced early-life violence: parental abandonment, her mother’s suicide, and her own suicide attempts.
- In later years, she faced recurrent respiratory issues overlooked due to her psychiatric background.

**Gender Conditioning:**

- Perceived as “emotional” and “exaggerating,” physical symptoms were minimized, delaying the advanced lung cancer diagnosis.
- Respiratory difficulties were dismissed as secondary to mental health, resulting in a delayed cancer diagnosis.

**Social Context:**

- Lack of social support and healthcare barriers led to the progressive decline in her physical and mental health.
- Lung cancer with vertebral metastasis was diagnosed after multiple admissions, highlighting how her psychiatric condition delayed the oncological diagnosis.

**Diagnostic Evolution:**

- After 17 years in a psychiatric facility for schizophrenia, she developed respiratory symptoms that were inadequately explored, leading to a delayed lung cancer diagnosis, followed by her passing after palliative care.

**Conclusions:**

This case highlights the need to consider gender-based biases in diagnosing women with mental health conditions. Her psychiatric history and gender contributed to the downplaying of her symptoms, delaying the crucial lung cancer diagnosis. Gender and mental health stigma can cause serious healthcare delays. A gender-sensitive approach is essential to ensure timely, thorough medical evaluations for women. Integrated mental and physical health care can prevent delays in diagnosis and improve outcomes for women with psychiatric disorders.

**Disclosure of Interest:**

None Declared

